# Association of Household and Community Characteristics with Adult and Child Food Insecurity among Mexican-Origin Households in *Colonias *along the Texas-Mexico Border

**DOI:** 10.1186/1475-9276-10-19

**Published:** 2011-05-13

**Authors:** Joseph R Sharkey, Wesley R Dean, Cassandra M Johnson

**Affiliations:** 1 Program for Research in Nutrition and Health Disparities, School of Rural Public Health, College Station, TX, USA; 2 Texas Nutrition and Obesity Policy Research and Evaluation Network Collaborating Center, Center for Community Health Development, School of Rural Public Health, College Station, TX, USA

## Abstract

**Background:**

Food insecurity is a critical problem in the United States and throughout the world. There is little published data that provides insights regarding the extent and severity of food insecurity among the hard-to-reach Mexican-origin families who reside in the growing *colonias *along the Texas border with Mexico. Considering that culture, economics, and elements of the environment may increase the risk for food insecurity and adverse health outcomes, the purpose of this study was to examine the relation between household and community characteristics and food insecurity.

**Methods:**

The study used data from the 2009 *Colonia *Household and Community Food Resource Assessment (C-HCFRA). The data included 610 face-to-face interviews conducted in Spanish by *promotoras *(indigenous community health workers) in forty-four randomly-identified *colonias *near the towns of Progreso and La Feria in Hidalgo and Cameron counties along the Texas border with Mexico. C-HCFRA included demographic characteristics, health characteristics, food access and mobility, food cost, federal and community food and nutrition assistance programs, perceived quality of the food environment, food security, eating behaviors, and alternative food sources.

**Results:**

78% of participants experienced food insecurity at the level of household, adult, or child. The most severe - child food insecurity was reported by 49% of all households and 61.8% of households with children. Increasing levels of food insecurity was associated with being born in Mexico, increasing household composition, decreasing household income, and employment. Participation in federal food assistance programs was associated with reduced severity of food insecurity. Greater distance to their food store and perceived quality of the community food environment increased the odds for food insecurity.

**Conclusions:**

The Mexican-origin population is rapidly expanding; record numbers of individuals and families are experiencing food insecurity; and for those living in rural or underserved areas such as the *colonias*, the worst forms of food insecurity are an ongoing reality. The rates of households with adult and child food insecurity in this border area are alarming and among the highest reported. Clearly, systematic and sustained action on federal, state, and community levels is needed to reduce household, adult, and child food insecurity that integrates cultural tailoring of interventions and programs to address food and management skills, multi-sector partnerships and networks, expansion of food and nutrition assistance programs, and enhanced research efforts.

## Introduction

The term food insecurity, which refers to all aspects of food and nutrition insufficiency, insecurity, and hunger describes an inadequate quality and/or quantity of food at the household, adult and/or child levels, and is a critical problem in the United States [1-10]. Prior work establishes the relationship between food insecurity and poor physical and mental health [8], or as a significant predictor of chronic illness and adverse physical and mental health outcomes in adults [11]. Food insecurity among children is associated with diminished nutritional status, poor academic performance, health-related quality of life, and developmental problems [12-16]. Outcomes related to poor nutrition affect a substantial number of Latino children, who are more likely than African American or white children to have mental and oral health problems, and high rates of overweight and obesity [17]. Furthermore, the prevention and management of nutrition-related health problems, such as obesity, diabetes, and cardiovascular disease, are complicated by food insecurity [18-24].

Prior to 2006, household food security status was described as "food secure", "food insecure without hunger", and "food insecure with hunger" [23,25]. In 2006, "food insecure without hunger" was changed to "low food security" and "food insecure with hunger" became "very low food security" [23]. Nationwide, the prevalence of food insecurity (low or very low food security) in 2009 was 14.7% of households, 16.6% of individuals living in food insecure households, 21.3% of households with children, and 11.8% of households with food insecure children [23]. Most food insecure households occasionally experienced diminished food supplies; however, one-fourth of food insecure households and one-third of households with very low food security experienced frequent or chronic food insecurity, such as running out of food every month [23]. National surveys, such as the 1999 Current Population Survey (CPS), 2009 CPS, and NHANES III have consistently found that Hispanic/Latino households were at the greatest risk for food insecurity [23,26-29]. Subgroup analyses from the USDA supplement to the CPS revealed that rates of food insecurity were higher in Hispanic households (26.9%) than in African American (24.9%) and non-Hispanic white (11.0%) households [23]. For Hispanic households with food insecure children or with very low food security among children, the prevalence in 2009 was 18.7% and 2.5%, respectively. This rate was two percentage points greater than African American households with food insecure children and 2.8 times larger than the 7.6% of non-Hispanic white households with food insecure children [23]. Since 1996, the two-year national average for prevalence of food insecurity and very low food security increased from 11.3 in 1996-1998 to 13.5% in 2007-2009; at the same time, the prevalence in Texas, which was significantly greater than the national average, increased from 15.2% to 17.4% [23].

**Figure 1:**
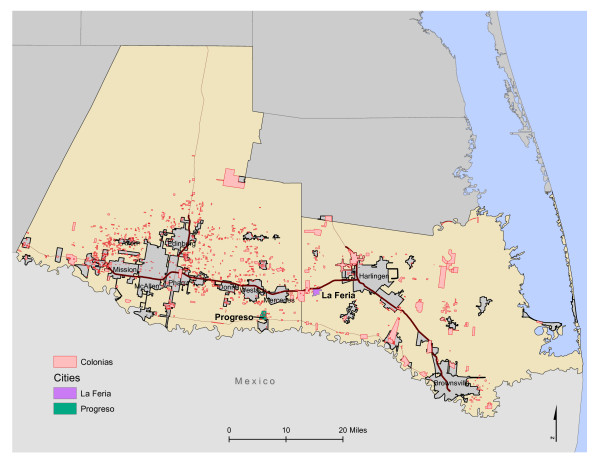
**Map of South Texas Border Region**.

According to 2009 estimates, persons of Hispanic origin comprised 15.8% of the U.S. total population and 36.9% of the population in Texas, which has the second largest percentage and number of Hispanic residents [30]. In Texas, the largest county-level percent of persons of Hispanic or Latino origin is along the Texas border with Mexico, where the percent exceeds 86% in each county [31]. The Texas border region is characterized by a Hispanic majority (predominately Mexican-origin) population, and above average number of Mexican-born immigrants. In this setting, residents are not as likely to have to choose between American and Mexican values, and most residents are Spanish-speakers [32,33]. The Texas-Mexico border region is one of the fastest growing areas of the United States, and estimates predict a doubling of the predominately Spanish-speaking population by 2025 [34]. Demands for low-cost housing along the Texas-Mexico border have resulted in the development of more than 2,294 *colonias*, a Spanish term that describes unincorporated settlements, neighborhoods, and communities, many lacking basic infrastructure such as paved roads, running water, or sewage [35,36]. In 2008, the population inhabiting Texas *colonias *was approximately 400,000 [36]. The burden of obesity and nutrition-related health conditions disproportionately affects marginalized populations that face increased vulnerability to food insecurity and poor nutritional health [37]. One, such marginalized population is Mexican-origin families who reside in impoverished *colonias *along the Texas-Mexico border [20]. Rates of nutrition-related health conditions, such as obesity and diabetes along the border are among the highest in the United States [38]. These families are considered one of the most disadvantaged, hard-to-reach minority groups in the United States [18]. In 2006, there were more than 1,786 *colonias *identified in the six most populous border counties in Texas, with a population of more than 350,000 [39]. Most of Texas' *colonias *are located in the South Texas border counties of Cameron and Hidalgo (see Figure 1), with about 60% of Texas' *colonias *located in Hidalgo County [40], which suffers from persistent poverty defined by at least 20% of the county falling below the poverty line for the period following the 1970 U.S. Census [41].

There is little published data that provides insights regarding the extent and severity of food insecurity among the hard-to-reach Mexican-origin families who reside in the growing *colonias *along the Texas border with Mexico [42]. One study of migrant and seasonal farmworkers found 82% with some experience of food insecurity during the previous 12 months (49% had very low food security) [43]. Considering that culture, economics, and elements of the environment may increase the risk for food insecurity and adverse health outcomes, the purpose of this study was to examine data from 610 face-to-face interviews conducted by *promotoras *(indigenous community health workers) in forty-four *colonias *near the towns of Progreso and La Feria in Hidalgo and Cameron counties along the South Texas border with Mexico to: 1) describe household characteristics and levels of household food insecurity, and 2) examine the relation between household and community characteristics and food insecurity.

## Methods

### Participants

The study used survey data of 610 adult women from the 2009 *Colonia *Household and Community Food Resource Assessment (C-HCFRA), which was conducted in 44 *colonias *near the towns of Progreso and La Feria in the Lower Rio Grande Valley of Texas from September to October 2009 (see Figure 1). After discussions with community partners and team *promotoras*, these two comparably sized communities were selected to examine household and community food resources; all protocols were approved by Institutional Review Board at Texas A&M University. According to 2009 data, 6,955 individuals live in La Feria (77.4% Hispanic and 29.2% of residents with income below poverty level) and 5,636 in Progreso (99% Hispanic and 50.9% with income below poverty level); each town is two to three square miles in size [44]. The *promotoras *drove the areas and enumerated the *colonias *using a "windshield survey"; trailer parks and *colonia*-like neighborhoods primarily occupied by "winter Texans" (seasonal residents who come for leisure and recreation) were excluded [45]. Equal numbers of *colonias *were included from each area.

### Data Collection

Four *promotoras *who had been involved in prior research projects in Hidalgo County underwent two days of training, which were conducted in Spanish and covered study purpose, door-to-door recruitment of participants, informed consent, survey administration, and disbursement of incentive at the completion of the survey. In addition to the training, the *promotoras *evaluated the Spanish version of the survey instrument for semantic, conceptual, and normative dimensions of equivalence, and for social and cultural appropriateness; and provided feedback for modifying the survey prior to data collection. After randomly identifying *colonias *from an enumerated list, a two-person bilingual, bicultural team of *promotoras *approached all residences, recruited participants and conducted face-to-face interviews in Spanish using a structured survey. One team was assigned to the Progreso area and the other to La Feria. Participants were identified by asking for an adult female who was responsible for household food acquisition and/or food preparation. All participants provided informed consent and received a $5 incentive for survey completion; each survey took approximately 20 minutes to complete. All data were collected in Spanish; all *promotoras *were native speakers. There were seven refusals in La Feria (response rate 97.3%; *n *= 248) and two in Progreso (response rate 99.4%; *n *= 362).

### Measures

The 2009 C-HCFRA included nine modules. *Demographic characteristics *were age, education, race/ethnicity, marital status, nativity, household composition (number of adults and children in the household), household income and frequency, and employment status (unemployed, employed part-time, and employed full-time). Federal poverty level (FPL) for 2009 was calculated from household income and composition data using 2009 Federal Poverty Guidelines [46]. *Health characteristics *included presence of diabetes or heart problems among any household resident, and self-reported height and weight, which were used to calculate body mass index (BMI) in kg/m^2^. Categories of BMI were constructed as normal (<25 kg/m^2^), overweight (25-29.9 kg/m^2^), and obese (≥30 kg/m^2^). *Access and mobility *were assessed through car ownership, availability and source of transportation, distance to main store for purchasing groceries, and names of primary and secondary stores utilized for grocery purchases. *Food cost *included the amount spent each week on groceries, date of last time to shop for groceries, and the amount spent during that trip. *Federal and community food and nutrition assistance programs *included four federal programs: 1) Supplemental Nutrition Assistance Program (SNAP), 2) Women, Infants, and Children (WIC), 3) School Breakfast Program (SBP), and 4) National School Lunch Program (NSLP); and assorted community and emergency food assistance programs such as food pantries, food banks and church programs. *Quality of food environment *assessed perceptions of community retail food sources using a 4-point Likert scale (e.g., 1 = strongly agree to 4 = strongly disagree) for three items about the local community: 1) little variety in the types of foods that can be purchased; 2) few grocery stores or supermarkets; and 3) food prices are high [47]. Perceptions related to the store where most of the groceries were purchased were assessed on a 5-point Likert scale (e.g., 1 = excellent to 5 = poor) using three questions: How would you rate 1) the variety, 2) the freshness, and 3) the price of fruits and vegetables at this store? Binary variables were constructed as fair/poor vs. all others [47].

*Food security *was measured using eleven items from the 12-item Radimer/Cornell measures of hunger and food insecurity that has been used in other Mexican-American populations to assess food anxiety, qualitative, and quantitative components of food insecurity on household, adult, and child levels [9,48,49]. Table 1 shows the four household, four adult, and three child items about which each participant was asked whether this was not true, sometimes true, or often true. Binary variables were constructed as often/sometimes true vs. never true. Four mutually exclusive categories of food security were constructed to represent the four-stage process as household food supplies are exhausted (e.g., food secure, household food insecure, adult food insecure, and child food insecure) [49]: Food secure households consisted of participants who answered not true to at least two items from each level (household, adult, and child); household food insecure individuals answered sometimes/often true to two or more household items and less than two items from adult and child levels; adult food insecure individuals answered sometimes/often true to at least two adult items and less than two child items; and child food insecure individuals responded sometimes/often true to at least two child items.

**Table 1:** Affirmative Responses to Food Security Items (*n *= 610)

	*Frequency of Occurrence*		
*Food security item*	Any (Sometimes or Often)	Often	Sometimes
***Household items***			
1 Worried that food would run out before I get money or food stamps to buy more	81.0	17.9	63.1
2 Food bought didn't last and I didn't have money to get more	73.6	12.6	61.0
3 We eat the same thing for several days in a row because we only have a few different kinds of food on hand and didn't have money to buy more	65.1	11.0	54.1
4 Ran out of food needed to put together a meal and didn't have money to get more	71.1	11.6	59.5
			
***Adult items***			
5 Can't afford to eat properly	61.8	10.2	51.6
6 Respondent hungry but didn't eat because couldn't afford enough food	58.5	10.0	48.5
7 Respondent ate less than he/she felt should because didn't have enough money for food	58.8	9.3	49.5
8 Adult(s) cut size or skipped meals last month because not enough money to buy food	60.0	10.7	49.3
			
***Child items***^*1*^			
9 Cannot give child(ren) a balanced meal because can't afford it	59.7	10.1	49.6
10 Child(ren) not eating enough because can't afford enough food	53.7	8.9	44.8
11 Child(ren) are hungry sometimes, but can't afford more food	51.0	8.5	42.6

^1 ^Included only households with children (*n *= 484)

*Eating behaviors *were measured by self-reported daily servings of fruit, vegetables, sugar-sweetened beverages, beans, and lean protein (e.g., fish and chicken), weekly frequency of fast-food meals and a regular breakfast meal. Two questions from a validated, self-reported two-item screener were combined to describe fruit and vegetable intake [50,51]. Validated measures from prior community-based work in North Carolina assessed consumption of sugar-sweetened beverages, frequency of fast food meals, and frequency of eating a regular breakfast meal [52-54].

*Alternative food sources *included the purchase of prepared food from neighbors or friends, mobile food vendors, and *pulgas *(flea markets).

### Analysis

Release 11 of Stata Statistical Software was used for all statistical analyses; *p *<0.05 was considered statistically significant. Descriptive statistics were estimated for food security items, as well as for demographic characteristics, health characteristics, access and mobility, quality of food environment, eating behaviors, and alternative food sources by food security status. A nonparametric χ^2 ^test for trend across ordered groups of food security status was performed. A conservative Bonferroni correction (alpha rejection region/number of tests to be conducted) was used to reduce Type I error rate for each individual test from 0.05 to 0.002 and 0.001 [55]. Since the four-category dependent variable (food security level) was not ordinal, multinomial logit was used [56]. A multinomial logit regression model was estimated to determine the association of independent variables with food-security status. Variables for demographic characteristics, food store access, perceived quality of food environment, alternative food sources, and eating behaviors were simultaneously entered; backward elimination strategy was used, which sequentially removed statistically non-significant variables, to obtain the "best" set of independent variables [55]. Adjusted coefficients, SE, and odds ratio (OR) are reported.

## Results

Table 1 presents frequencies for affirmative responses to each of the household, adult, and child food security items. At the household level, 81% experienced food anxiety (item 1), 65% limited quality (item 3), and limited quantity (items 2 and 4). Limited quality at adult level (item 5) was reported by 61.8% and limited quantity (items 6-8) by more than 58%. At the child level, 59% reported limited quality (item 9) and at least 51% experienced limited quantity (items 10-11). In data not shown in Table 1, 59.5% (*n *= 363) of households experienced all four items; 49.7% (*n *= 303) answered affirmatively to all four adult items. Among the households with children, 48.8% (*n *= 236) responded positively to all three child items.

More than three-quarters of participants (78%) experienced food insecurity at the level of household, adult, or child; 22.1% of households were classified food secure. The most severe - child food insecurity was reported by almost half (49%) of all households and 61.8% of households with children. Table 2 presents demographic and health characteristics by food security status. Most of the participants described themselves as Mexican rather than Mexican-American; 67.7% were born in Mexico; 60% were married, 79.3% had at least one child under the age of 18 residing with them (ages ranged from 1 month to 17 years), and most were unemployed (respondent and/or spouse). Almost 15% of households with children were single parent. Almost 97% of 455 households who reported income had household incomes at or below 100% FPL; 85.7% at or below 75% FPL. A positive trend across increasing levels of food insecurity was observed for participants who were born in Mexico, completed <7 years of education, lived with households with a greater number of adults and children, reported a household 100% FPL or 75% FPL, and worked full-time. About 54% of households with children participated in federal school nutrition programs; participation was significantly lower among families with more severe levels of food insecurity. More than 37% of households (*n *= 227) did not participate in any Federal food assistance and nutrition programs; 44.9% did not participate in SNAP, and 57.2% and 45.9% of households with children did not participate in WIC or NSLP, respectively. Community-based emergency food sources such as a food bank or church were used by 3.1% (*n *= 19) of households. For the 55% who received SNAP benefits, their monthly benefits lasted fewer days with increasing levels of food insecurity. Thirteen of the trends remained significant after adjusting for multiple comparisons with a revised level of statistical significance (*p *≤ 0.002)

**Table 2:** Description of Participants' Demographic and Health Characteristics by Food Security Status

	All (*n *= 610)	Food Secure (*n *= 135)	Household Insecure (*n *= 74)	Adult Insecure (*n *= 102)	Child Insecure (*n *= 299)
***Demographic characteristics***					
Age^1^	39.9 ± 14.4	38.6 ± 13.8	36.9 ± 13.0	43.9 ± 18.4	39.8 ± 13.1
	(37)	(36)	(35.5)	(40)	(38)
Race/ethnicity					
Mexican	61.8	48.1	55.4	64.7	68.6***^†^
Mexican American	27.5	37.0	20.3	26.5	25.4*
Marital status					
Married	60.0	58.5	63.5	51.0	62.9
Country of birth					
Mexico	67.7	51.1	60.8	73.5	74.9***^†^
Education^2^					
<7^th ^grade	31.8	20.6	22.2	35.0	38.2***^†^
7^th^-11^th ^grade	32.8	29.8	38.9	40.2	30.2
High school graduate	35.4	49.6	38.9	24.7	31.6***^†^
Household composition^1^					
Adults	1.9 ± 0.7	1.9 ± 0.6	1.9 ± 0.6	1.7 ± 0.6	2.0 ± 0.7*
	(2)	(2)	(2)	(2)	(2)
Children^3^	2.5 ± 1.4	2.2 ± 1.3	2.4 ± 1.3	2.7 ± 2.2	2.6 ± 1.3**
	(2)	(2)	(2)	(2)	(2)
Total	3.9 ± 1.8	3.7 ± 1.7	4.1 ± 1.6	3.1 ± 2.3	4.3 ± 1.7***^†^
	(4)	(4)	(4)	(2)	(4)
Single parent household^3^	14.7	15.6	13.6	18.0	13.9
Poverty status^4^					
Income data not	25.4	24.4	28.4	19.6	27.1
reported					
≤ 75% FPL	63.9	50.4	62.2	66.7	69.6***^†^
76%-100% FPL	8.4	19.3	6.8	10.8	3.0***^†^
>100% FPL	2.3	5.9	2.7	2.9	0.3***^†^
Employment status					
Female					
Unemployed	51.5	48.9	71.6	58.8	45.1
Part-time	22.0	18.5	12.2	18.6	27.1
Full-time	26.6	32.6	16.2	22.5	27.8**
Male					
Unemployed	60.0	49.6	39.2	71.6	65.9***^†^
Part-time	14.3	8.1	27.0	18.6	12.4
Full-time	25.7	42.2	33.8	9.8	21.7***^†^
Food assistance program					
SNAP	55.1	45.2	74.3	52.9	55.5
Days SNAP benefits	20.1 ± 8.0	22.8 ± 8.1	21.0 ± 7.6	19.8 ± 8.9	18.8 ± 7.6***^†^
last^5^	(21)	(21)	(21)	(21)	(21)
WIC^3^	42.8	35.8	57.6	50.0	40.5
School breakfast^3^	53.9	68.8	69.7	42.0	45.9***^†^
School lunch^3^	54.1	68.8	69.7	42.0	46.3***^†^
***Health characteristics***					
Diabetes	24.6	26.7	18.9	25.5	24.7
Body mass index (kg/m^2^)^6^					
Normal (<25)	30.3	40.2	26.0	32.3	26.4**
Overweight (25-29.9)	35.0	31.5	37.0	33.3	36.6
Obese (≥ 30)	34.7	28.3	37.0	34.3	36.9

^1 ^Data presented as mean ± SD (median)

^2 ^Education data provided by 588 participants

^3 ^Data based on 484 households with children residing in the household

^4 ^Household income data provided by 455 participants

^5 ^Mean ± SD (median) based on those who received SNAP benefits

^6 ^Height and weight data provided by 594 participants

Statistical significance in trend across categories of increasing food insecurity: *p≤0.05 **p≤0.01 ***p≤0.001

^† ^Statistically significant after Bonferroni adjustment for multiple comparisons *p *≤ 0.002

Table 3 describes participants' access and mobility, quality of food environment, eating behaviors, and alternative food sources by food security status. On average, participants travelled 10 miles one-way to purchase most of their groceries; only 17 participants (2.8%) shopped for groceries in their town (data not shown); 75% of main food stores are a supermarket, supercenter, or mass merchandiser; and almost 63% of participants purchased groceries at least once a week. The use of supermarkets as the main food store declines with increasing levels of food insecurity. Significant difference by food-insecurity level was found for less favorable perceptions of community food resources and food stores utilized, greater weekly consumption of beans and a regular breakfast meal, and less reliance on neighbors, friends, or *pulgas *(flea markets) for prepared foods. Eleven of the variables remained statistically significant after Bonferroni adjustment (*p *≤ 0.001) overall, 24.9% of participants purchased prepared foods from a neighbor or friend, 29.7% from a mobile food vendor, and 30.7% from a *pulga*. The main items purchased from mobile food vendors were ice cream (27.9%), *raspas *or shaved ice (8.4%), and *elotes *or roasted corn on the cob or in a cup (8.2%). Participants purchased the following food items from the *pulgas*: fresh fruit and vegetables (20.5%), *aguas frescas *or sugar-sweetened fruit waters (8.5%), *raspas*, *elotes*, tacos, Mexican soft drinks, tamales, and *menudo *(traditional Mexican soup).

**Table 3:** Description of Participants' Access and Mobility, Quality of Food Environment, Eating Behaviors, and Alternative Food Sources by Food Security Status

	All (*n *= 610)	Food Secure (*n *= 135)	Household Insecure (*n *= 74)	Adult Insecure (*n *= 102)	Child Insecure (*n *= 299)
***Access and mobility***					
Own car	70.7	77.0	73.0	62.7	69.9
Car available during day	69.2	68.1	62.2	67.6	71.9
Other transportation					
Friend	8.2	8.1	2.7	9.8	9.0
Neighbor	23.8	8.1	10.8	25.5	33.4***^†^
Relative	63.4	37.0	33.8	67.6	81.3***^†^
Charge for transportation^1^	14.8	10.9	25.9	11.5	15.4
Store where buy most of groceries					
Distance^2^	10.0 ± 2.2	9.3 ± 2.8	9.8 ± 1.5	10.2 ± 2.7	9.6 ± 3.0
	(10)	(10)	(10)	(10)	(10)
Ride with friend or family	29.5	21.5	27.0	39.2	30.4
Type of main store					
Supermarket	62.3	74.1	78.4	61.8	53.2***^†^
Supercenter or mass merchandiser	12.9	11.8	10.8	12.7	14.0
Frequency					
≥ 1 time/week	62.9	71.1	50.0	59.8	63.5
Every two weeks	27.5	17.0	44.6	27.4	28.1
Type of 2^nd ^store					
Supermarket	15.3	7.4	20.3	13.7	18.1*
Small grocery store	13.4	26.7	24.3	10.8	5.7***^†^
Supercenter or mass merchandiser	63.4	52.6	50.0	70.6	69.2***^†^
Frequency					
≥ 1 time/week	61.1	58.5	43.2	61.8	66.6**
Every two weeks	26.6	23.7	36.5	28.4	24.7
Weekly expenditures for	95.3 ± 56.8	91.7 ± 54.8	97.0 ± 47.1	88.4 ± 58.5	98.8 ± 59.1
groceries^3^	(80)	(80)	(100)	(75)	(80)
***Quality of food environment***					
Community food resource					
Little variety in types of foods	92.5	86.7	91.9	93.1	95.0**
Few grocery stores or supermarkets	93.1	89.6	90.5	93.1	95.3*
Food prices are high	94.4	88.9	94.6	95.1	96.7**
Store where purchase most of groceries					
Fair-to-poor variety of fruits and vegetables	10.2	8.1	14.9	7.8	10.7
Poor freshness of fruits and vegetables	11.3	5.9	8.1	8.8	15.4**
Poor quality of fruits and vegetables	17.9	7.4	18.9	17.6	22.4***^†^
Spotty	5.4	2.2	6.8	2.9	7.4*
Soft	7.4	3.0	8.1	8.8	8.7
Overripe	13.9	4.4	10.8	11.8	19.7***^†^
Fruits and vegetables expensive	29.0	18.5	45.9	33.3	28.1
***Eating behaviors***					
Daily servings of fruit	1.9 ± 0.9	2.0 ± 1.1	1.6 ± 0.7	2.0 ± 0.8	1.9 ± 0.9
	(2)	(2)	(2)	(2)	(2)
Daily servings of vegetables	1.5 ± 0.9	1.6 ± 1.1	1.6 ± 1.0	1.6 ± 0.9	1.5 ± 0.9
	(1)	(2)	(1.5)	(1)	(1)
Daily servings of sugar-sweetened beverages	1.7 ± 1.6	1.7 ± 1.8	1.6 ± 1.5	1.8 ± 1.8	1.7 ± 1.5
	(1)	(1)	(1)	(1)	(1)
Fast food meals					
Weekly frequency at	1.1 ± 1.1	1.4 ± 1.5	0.7 ± 0.9	1.0 ± 1.1	1.0 ± 0.9
fast food restaurant	(1)	(1)	(1)	(1)	(1)
Weekly frequency	0.7 ± 0.9	0.8 ± 1.1	0.5 ± 0.8	0.8 ± 1.1	0.8 ± 0.9
bring home to eat	(0)	(0.5)	(0)	(0)	(1)
Weekly frequency eat	5.0 ± 3.2	3.7 ± 2.3	4.1 ± 2.5	5.7 ± 3.8	5.6 ± 3.2***^†^
pinto or black beans	(5)	(3)	(3.5)	(7)	(7)
Weekly frequency eat	3.2 ± 2.8	2.7 ± 1.2	3.0 ± 1.7	3.8 ± 4.0	3.3 ± 2.9
chicken or fish	(3)	(3)	(3)	(3)	(3)
Weekly frequency of	4.5 ± 2.9	4.2 ± 2.8	3.9 ± 2.7	5.4 ± 2.4	5.4 ± 2.4***^†^
regular breakfast meal	(7)	(4)	(3)	(7)	(7)
***Alternative food sources***					
Purchase prepared food from a neighbor or friend	24.9	45.9	25.7	17.6	17.7***^†^
Purchase food from mobile food vendors	29.7	29.6	20.3	25.5	33.4
Purchase food from *pulga *(flea market)	30.7	43.7	31.1	22.5	27.4***^†^

^1 ^Proportion of participants who receive transportation from friend, neighbor, or relative (*n *= 420)

^2 ^Data provided by 592 participants.

^3 ^Data provided by 604 participants

Statistical significance in trend across categories of increasing food insecurity: *p≤0.05 **p≤0.01 ***p≤0.001

^† ^Statistically significant after Bonferroni adjustment for multiple comparisons *p *≤ 0.001

Table 4 (adjusted multinomial logit regression estimates) shows the characteristics that increased the odds for household, adult, and child food insecurity (compared with food secure). Demographic characteristics were independently associated with increasing levels of adult and child food insecurity; namely, being born in Mexico, increasing household composition, household income, and employment. Interestingly, households that did not report an income were more likely to be child food insecure. Participation in federal food assistance programs was associated with lower severity of food insecurity. SNAP participants were more likely to report household food insecure; households where children participated in the NSLP were more likely to be food secure compared with food insecure. Greater distance to the food store where most of groceries were purchased increased the odds for adult food insecurity; items that described perceived quality of the community food environment were associated with household or child food insecurity levels. Interestingly, the odds for adult or child food insecurity were lower for participants who utilized alternative food sources. Households that purchased prepared foods from a neighbor or friend were more likely to be food secure.

**Table 4:** The Influence of Demographic Characteristics, Food Store Access, Perceived Quality of Community Food Environment, Alternative Food Sources, and Eating Behaviors on Food Security, Using Multinomial Logit Estimates for Household, Adult, and Child Food Insecure as Compared to Food Secure for Participants

	*Household*		*Adult*		*Child*	
	*Food Insecure*		*Food Insecure*		*Food Insecure*	
			
***Independent Variables***	Coefficient	OR^1^	Coefficient	OR^1^	Coefficient	OR^1^
	(SE)		(SE)		(SE)	
***Demographic characteristics***						
Nativity (Mexico-born)	0.102	1.11	0.931**	2.54	0.758**	2.13
	(0.339)		(0.337)		(0.258)	
Household composition	-0.072	0.93	-0.179	0.84	0.244**	1.28
	(0.119)		(0.118)		(0.086)	
Household income^2^						
≤75% FPL	0.245	1.28	0.796*	2.22	0.341	1.41
	(0.385)		(0.377)		(0.288)	
76%-100% FPL	-0.321	0.72	0.139	1.15	-1.240**	0.29
	(0.626)		(0.553)		(0.500)	
>100% FPL	-0.033	0.97	-0.221	0.80	-2.275*	0.10
	(0.976)		(0.843)		(1.113)	
Employment status^3^						
Spouse or partner	-0.090	0.91	1.497***	4.47	1.374***	3.95
unemployed	(0.379)		(0.437)		(0.289)	
Spouse or partner	1.414**	4.11	2.328***	10.26	1.055**	2.87
employed part-time	(0.482)		(0.545)		(0.423)	
***Food assistance programs***						
SNAP participant^4^	1.085**	2.96	0.588	1.80	-0.012	0.99
	(0.363)		(0.324)		(0.255)	
School lunch program	0.025	1.03	-1.566***	0.21	-0.764**	0.47
	(0.361)		(0.365)		(0.269)	
***Food store access***						
Distance to store where	0.073	1.08	0.177**	1.19	0.089	1.09
most groceries are purchased	(0.070)		(0.059)		(0.047)	
***Quality of food environment***						
Little variety in types of	0.952	2.59	0.682	1.98	1.200**	3.32
foods in community	(0.561)		(0.540)		(0.416)	
Fruits and vegetables	-0.005	0.99	0.710	2.03	1.109**	3.03
not fresh where shop	(0.625)		(0.581)		(0.473)	
Fruits and vegetables	1.219***	3.38	0.802*	2.23	0.364	1.44
expensive where shop	(0.362)		(0.351)		(0.295)	
***Alternative food sources***						
Purchase prepared food	-0.425	0.65	-1.065**	0.34	-1.094***	0.33
from neighbor or friend	(0.386)		(0.383)		(0.283)	
***Eating behaviors***						
Weekly frequency of	-0.395*	0.67	-0.055	0.95	0.024	1.02
fast-food meals	(0.179)		(0.140)		(0.111)	

(*N *= 610) χ^2 ^(df = 45) = 279.76 Pseudo R^2 ^= 0.185						

^1 ^Odds ratio

^2 ^Referent group: participants who did not provide income data (*n *= 155)

^3 ^Referent group: spouse or partner employed full-time

^4 ^SNAP = Supplemental Nutrition Assistance Program (formerly Food Stamp Program) Statistically significant: *p≤0.05** *p*≤0.01***p≤0.001

## Discussion

Healthful nutrition, which depends on a sufficient household food supply, is vital to health in adults and to academic performance and development in children [8,11-16,18-20]. Considering the importance of access to an adequate quality and quantity of food among disadvantaged populations who may be more at risk for nutrition-related health problems [33,57-59], few studies have focused on the extent of food resource vulnerability among the growing Mexican-origin population [16,49,60-63]. Of these, only one examined the extent and correlates of increasing levels of severity of food insecurity among the rapidly growing Mexican-origin population along the Texas border with Mexico [43]. Although there are slight differences between the Radimer/Cornell measure of food insecurity and the Current Population Survey, the emerging picture of food insecurity among hard-to-reach Mexican-origin families suggests greater prevalence of adult and child food insecurity than the previously reported national, regional, and local rates among Hispanic adults and children [16,23,26,49,60-64]. This study extends our understanding of levels of food insecurity: household, adult, and child [9]. This is the first study, to our knowledge, that examines the relationship between nine components of household and community characteristics to levels of food security status among *colonia *residents. These components include demographic characteristics, health characteristics, access and mobility, food cost, federal and community food and nutrition assistance programs, perceived quality of the food environment, food security, eating behaviors, and alternative food sources.

Our analyses revealed that national data on the prevalence of food insecurity among Hispanic households underestimates the prevalence and severity of food insecurity among Mexican-origin families in border communities. Findings should be considered in context of the high rates of obesity and diabetes prevalent in these areas [33]. The 2009 report on household food insecurity among Hispanic households, which included Hispanics regardless of country of origin (e.g., Mexico, Puerto Rico, Cuba), identified 26.9% of households with low or very low food security [23]. Our analyses revealed that 78% of 610 *colonia *households experience some level of food insecurity. Specifically, data indicated 12.1% of respondents were household food insecure, 16.7% were adult food insecure, and 49% of households (61.8% of households with children) were at the most severe level of household food insecurity; that is, households with children who were food insecure. The overall prevalence of food insecurity observed in this study is three times that of the most recent national study [23]. Further, the very high level of severe food insecurity observed (49%) is much greater than the 27% observed in a sample of 211 Mexican American families in California [49], the 3.4% observed among 559 low-income Latino women [62], 14.8% among 256 low-income Latino families [61], or 1.6% among Hispanic mothers in northern California [16]. None of these reports appears to describe the more vulnerable Mexican-origin adults or children who reside in border areas. There is one study of 100 migrant and seasonal farm worker families in border areas of Texas and New Mexico that found a similarly high prevalence of food insecurity where 82% experienced some degree of food insecurity and 49% food insecurity with hunger [43]. Although the prevalence of more severe food insecurity in our sample is unacceptably large, it may understate the "true" prevalence among *colonia *households. Abarca describes a group of working-class Mexican and Mexican-American women residing along the Texas-Mexico border as cooks-as-artists, who demonstrated creativity and culinary expertise in their everyday food practices [65]. For instance, one woman, who did not have a sink and used a one-burner portable stove for cooking, was able to overcome limitations to create delicious meals for her family. It may be that respondents in the present study do not perceive "a lack" of food or other resources because they see themselves as creative agents who are able to provide sufficient food for their families.

In addition to 49% of this study's sample living in households with child food insecurity, the findings on socioeconomic disadvantage were disturbing. Unemployment rates were quite high; 60% of male spouses or partners were unemployed, and only14% worked part-time. Almost 15% of households with children were single-parent. Household income was extremely low; 64% reported a household income at or below 75% FPL and only 2.3% reported an income greater than 100% FPL. Food assistance program participation was low, given the very low household incomes; 45% did not receive SNAP benefits, and 46% of households with children did not participate in the National School Lunch Program (NSLP). These rates are somewhat higher for SNAP participation and lower for NSLP participation than noted in an urban sample of 320 Latinos (70% Mexican) where 30% reported household food insufficiency; 30% were Food Stamp participants; and 90% of children received school meals [26]. The participation of border *colonia *households in SNAP and NSLP was lower than noted in the most recent national report on 2009 estimates of household food insecurity in the United States, which combined all households regardless of race/ethnicity, and found that 30.8% of all households with an income less than 130% FPL and food insecure did not receive SNAP benefits in the previous 12 months; and 27.7% of households with an income less than 185% FPL and school-age children in the household did not receive a free or reduced-price lunch in the previous 30 days [23]. The use of alternative food sources, such as sale of prepared foods by neighbors or friends, mobile food vendors, and *pulgas *(flea markets), especially in areas without ready access to retail food stores or reliable transportation, is underreported [20,66]. This also is apparently the first study to identify the use of alternative food sources by *colonia *residents along the border. Overall, 24.9% of the sample purchased prepared foods from a neighbor or friend; 17% among the more food insecure and 45.9% among food secure households. Almost 30% purchased food items from mobile food vendors that marketed in their neighborhood. More than 30% (43.7% of food secure households) purchased food from *pulgas*, which are known to sell a wide variety of inexpensive fresh fruit and vegetables and prepared foods [66]. These findings suggest that further research should be conducted on the relationship between acquisition oriented coping strategies and food security.

Several additional findings from the adjusted multinomial logit regression model warrant mention. First, the results suggest that several household and community characteristics increased the odds for adult and child food insecurity; namely, being Mexico-born, increasing number of adults and children in the household, income ≤ 100% FPL, and unemployed spouse or partner. Others have linked food insecurity among Hispanics with low household incomes [49,63], minor children in the home and larger households [43], and households occupied by Mexican-born immigrants [43]. In a study of 630 Latino and Asian legal immigrants in urban areas of California, Texas, and Illinois, researchers found the following characteristics associated with being food insecure with hunger: household income below 100% FPL, receipt of food stamps (now SNAP), and being Latino [67]. Although other studies found the perception of diminished variety and quality of foods was associated with lower fruit and vegetable consumption, this is apparently the first study to link these perceptions to food insecurity [47,68]. This suggests that food insecure households in the border *colonias *face challenges from being located in disadvantaged neighborhoods, where there is limited or non-existent ready access to large supermarkets [20], and the stores that are accessible market a less desirable variety and quality of food items, especially fruit and vegetables [47]. Second, participation in two of the largest federal food and nutrition assistance programs were associated with a lower burden of food insecurity. Although households that participated in SNAP were more likely to be household food insecure compared with food secure, there was no association with adult or child levels of food insecurity. This suggests that greater participation in SNAP may provide enough resources to reduce the severity of food insecurity in this population [69]. However, *colonia *households with child food insecurity were more likely to exhaust SNAP benefits earlier than other households. With regards to NSLP, participation increased the odds for a household being food secure, compared with adult or child food insecure. As others have observed, there is an apparently large gap between nutritional need and utilized nutrition services [67]. Third, households with adult or child food insecurity were less likely than food secure households to use alternative food sources, such as purchasing prepared food from a neighbor or friend, or from a *pulga*, perhaps because these households were financially constrained and preferred reciprocity-based food acquisition systems over purchasing food from others. Although we do not have the data to support this, plausible explanations for limited reliance on these alternative food resources could include neighborhood variation in the availability of *pulgas *and friends and neighbors who sell food from their homes. A family's social capital, their relationship with friends, neighbors, and others within a community, may also impact their ability to access community resources including small food businesses run from neighborhood homes [52,70]. Few *colonias *are located within walking distance of a pulga, so the availability of transportation may also play a role. Finally, we do not fully understand why households with incomes ≤ 75% FPL are food secure; given very low income and lower participation in SNAP. The creative capacity and expertise observed in the culinary practices of Mexican and Mexican American women and documented by Abarca and Dean and colleagues suggests that economically-constrained women may be able to successfully mitigate challenging circumstances in order to provide sufficient food for their families [65,71]. Another explanation may be reliance on meals from neighbors, friends, or family. In addition, literature highlights how women use food to create and strengthen relationships with other women [65,72]. Extremely low income women may exchange food with female neighbors, friends, or family members as a means to maintain food security in their household. Unfortunately, there were no survey items assessing this type of interaction within social networks. It is worth noting that little literature exists elucidating the strategies that low-income Mexican-origin women use in food choices, much less to overcome hardships associated with food insecurity [73].

There are several major strengths to this study, especially in relation to other studies of food insecurity in Hispanic/Latino populations. This study is one of a few studies that collected data from a largely Mexican-origin region of the United States [19,32,33,43,45]; specifically from two difficult to access border areas that demonstrated high nutritional need. The first is the development of Household and Community Food Resource Assessment (C-HCFRA) survey and data collection approaches in collaboration with team *promotoras *to consider culture, language, trust, and cognitive demands of Mexican-origin residents who live in border *colonias*. The second is the delivery of the survey by trained *promotoras *who are indigenous community health workers, native Spanish-speakers, knowledgeable of the communities, and trusted by *colonia *residents. As a result, the participant recruitment and survey completion rate was an extremely high 98.5%, which was greater than previously reported in urban border areas [32].

The study has several limitations. Data were not available on acculturation or immigration experiences as identified by others [33], or on documentation status. Documentation status was not asked of participants due to its sensitivity. Another limitation is lack of income data on 25% of participants. Additionally, the cross-sectional nature of the data prevents an examination of causality in severity of household food insecurity. Confirmation of these findings in other border *colonia *areas is necessary. Finally, the use of the Radimer/Cornell measure of food insecurity limits our ability to compare accurately prevalence with national data.

## Conclusions

Despite these limitations, these findings are both timely and indispensible. Currently in the United States, the Mexican-origin population is rapidly expanding; record numbers of individuals and families are experiencing food insecurity nationwide; and for those living in rural or underserved areas such as the *colonias*, food insecurity is an ongoing reality for many adults and children. The rates of households with adult and child food insecurity in this border area are alarming and among the highest reported. Unfortunately, a large percentage of households that lack quality and quantity of food include children, which is especially troubling given the importance of good nutrition on optimal growth, function, and health [67]. Young children of Mexican immigrant families have a greater risk for hunger and household food insecurity [64], and are less likely to meet dietary recommendations than other children [49,61]. In addition, the population in the *colonias *is burdened by high rates of diet-related chronic diseases and home to a disparate gap between nutritional need and nutritional resources. Considered together, the results suggest that a large proportion of families living in the *colonias *are facing adult and child food insecurity and potentially at risk for adverse health outcomes across the life course. This paper therefore provides compelling evidence for enhanced research efforts that will lead to better understanding of coping strategies and the use of federal and community food and nutrition assistance programs for reducing hardship associated with food insecurity. Clearly, systematic and sustained action on federal, state, and community levels is needed to reduce household, adult, and child food insecurity that integrates cultural tailoring of interventions and programs to address food and management skills, multi-sector partnerships and networks, expansion of food and nutrition assistance programs, and enhanced research efforts [10,74].

## Competing interests

The authors declare that they have no competing interests.

## Authors' contributions

JRS developed the original idea for the community assessment. JRS and WRD worked on the development of the instrument and the protocol for collection of data. JRS wrote the first draft of the paper. JRS, CMJ, and WRD read and approved the final manuscript.
